# Peptide Arrays as Tools for Unraveling Tumor Microenvironments and Drug Discovery in Oncology

**DOI:** 10.3390/cells15020146

**Published:** 2026-01-14

**Authors:** Anna Grab, Christoph Reißfelder, Alexander Nesterov-Mueller

**Affiliations:** 1 Department of Surgery, Mannheim Medical Center, Ruprecht-Karls-University Heidelberg, 68167 Mannheim, Germany; 2 Institute of Microstructure Technology, Karlsruhe Institute of Technology (KIT), Hermann-von-Helmholtz-Platz 1, 76344 Eggenstein-Leopoldshafen, Germany; alexander.nesterov-mueller@kit.edu

**Keywords:** tumor microenvironment, functional peptide library, drug screening

## Abstract

**Highlights:**

**What are the main findings?**

- Recent achievements report on the application of peptide array technology in the study of tumor microenvironment.

**What are the implications of the main findings?**

- Peptide chip mapping of tumor microenvironment interactions can help identify metastasis-related biomarkers.
- Peptide arrays enable drug screening for personalized therapies.

**Abstract:**

Peptide arrays represent a powerful tool for investigating a wide application field for biomedical questions. This review summarizes recent applications of peptide chips in oncology, with a focus on tumor microenvironment, metastasis, and drug mechanism of action for various cancer types. These high-throughput platforms enable the simultaneous screening of thousands of peptides. We report on recent achievements in peptide array technology for tumor microenvironments, an enhanced ability to decipher complex cancer-related signaling pathways, and characterization of cell-adhesion-mediating peptides. Furthermore, we highlight the applications in high-throughput drug screenings for development of immune therapies, e.g., the development of novel neoantigen therapies of glioblastoma. Moreover, epigenetic profiling using peptide arrays has uncovered new therapeutic targets across various cancer types with clinical impact. In conclusion, we discuss artificial intelligence-driven peptide array analysis as a tool to determine tumor origin and metastatic state, potentially transforming diagnostic approaches. These innovations promise to accelerate the development of precision cancer approaches.

## 1. Introduction—Peptide Arrays as Decoders of Tumor Complexity

The global incidence of cancer is rising worldwide, emphasizing the need for a deeper understanding of the underlying molecular mechanisms. To investigate tumor initiation and the development of therapeutic resistance, a wide range of biological parameters must be considered, including genomic alterations, clonal evolution, cell–cell interactions, angiogenesis, signaling pathways, and protein networks [1,2,3,4,5]. Analyzing these complex systems requires advanced high-throughput technologies capable of generating and processing large-scale datasets. Various analytical methods, such as peptide chips, enable the parallel detection of numerous molecular interactions and thereby facilitate comprehensive data acquisition [6,7]. In genomic screenings, hundreds of thousands to millions of spots are typically required, whereas proteomic or peptide screenings often rely on tens to hundreds of thousands of spots to capture relevant interactions and sequence combinations [8,9]. Peptide arrays enable, through miniaturization and parallel analysis, a much higher degree of multiplexing and throughput than conventional single-target assays such as ELISA (e.g., 10–100× throughputs) [10]. They are more flexible to produce than mass spectrometry-based methods for identifying ligand-binding sites, neoantigen sequences [11], or broad tumor sample screening [12,13]. The intricate molecular and cellular heterogeneity of tumors continues to challenge conventional diagnostic and therapeutic strategies. Peptide chips enable those screenings, e.g., for dynamic cell–cell and cell–matrix interactions that can drive tumor progression and therapy resistance [14]. By enabling high-throughput and reproducible screening, peptide arrays overcome many limitations of conventional biochemical and cell-based assays. Their key advantages include (i) scalability and miniaturization, allowing simultaneous testing of thousands of interactions; (ii) minimal sample and reagent consumption; (iii) quantitative and spatially resolved data output; (iv) high reproducibility and automation compatibility; and (v) seamless integration with multi-omics and imaging approaches [15,16]. While high-throughput technologies such as single-cell sequencing [17,18], spatial transcriptomics, and mass-spectrometry-based proteomics have profoundly transformed cancer research, they predominantly provide correlative or inferential insights into cellular states rather than direct measurements of functional molecular interactions [19]. Transcriptomic and spatial approaches capture gene expression patterns [20] with high resolution in 2D and 3D [21], yet they often fail to reflect post-transcriptional regulation, protein activity, or transient signaling events that critically shape tumor behavior [22]. Similarly, proteomic workflows offer deep coverage but remain constrained by sample complexity, dynamic range issues [23], and difficulty in resolving weak, context-dependent interactions that are critical in tumor microenvironments and adaptive resistance mechanisms [24,25,26,27]. Within the tumor microenvironment, cell behavior is influenced by extracellular matrix cues, short linear motifs, post-translational modifications, and immunogenic epitopes that are poorly predicted by gene expression alone [28,29]. Peptide arrays address a complementary niche by enabling the direct interrogation of molecular binding events, such as receptor–ligand interactions and epitope recognition, and can discriminate distinct cancer states or perform scalable multi-disease profiling [30]. Depending on the biological question, we categorize assays with peptide arrays here in three principal domains: cell-based assays, blood-based assays, and tumor sample analyses. In cell-based assays, high-density peptide matrices enable upon the functionality the identification of short peptide motifs [14] governing cell adhesion, migration, and repulsion. The screenings can be multiplexed with capture molecules, e.g., DNA oligonucleotides [31], to reveal ligand–receptor interactions that regulate focal adhesion [32], integrin signaling, and cytoskeletal dynamics [33]. In blood-based applications, peptide arrays facilitate high-throughput profiling of circulating biomolecules such as autoantibodies [34], neoantigens [12,35], and cytokines [36]. In tumor-based assays, peptide and proteomic protein arrays provide a platform for analyzing tumor cell phenotype, immune recognition, and target identification [37] for drug development [38]. However, peptides face technical limitations such as higher-order protein structures and binding affinity, which need orthogonal methods to fully interpret biological relevance [30]. Peptide arrays bridge the gap between descriptive profiling and actionable functional readouts. Their scalability and adaptability position them not only as research tools, but also as potential components of routine diagnostics and personalized monitoring with growing market size [39]. In this review, we summarize the technological evolution and emerging applications of peptide array platforms in oncology.

## 2. Evolution and Application of Peptide Arrays in Cancer Research

**Figure 1:**
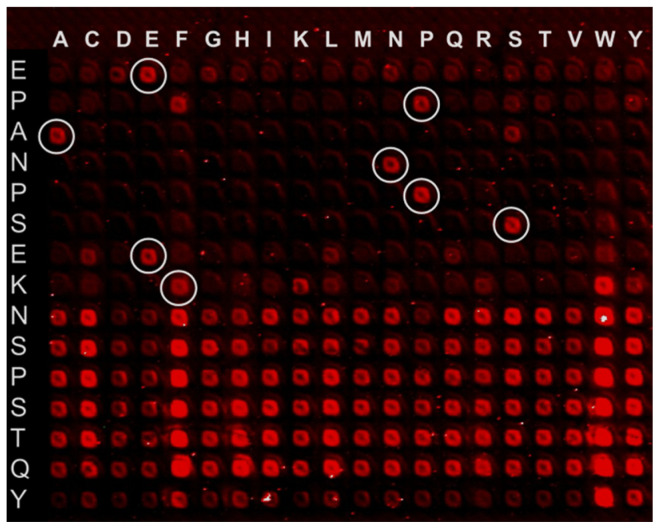
Fluorescence scan of the substitutional array for the cyclized peptide EPANPSEKNSPSTQY after incubation with rituximab and subsequent immunostaining. The white circles show amino acids from the original sequence EPANPSEK. The cyclic peptides were formed via the thioether macrocyclization [45].

Early generations of peptide microarrays [40,41] provided the first proof-of-concept for measuring enzyme activities and protein–protein interactions from tumor lysates, revealing early insights into oncogenic signaling and potential biomarkers. From around 2010 onward, high-density peptide arrays produced via electrical fields of high-voltage CMOS-based laser printing and robotic spotting [42,43,44] expanded spot densities to >10^4^ peptides cm^−2^, minimizing cross-contamination and allowing reliable quantification of low-abundance signaling events. One application is in the field of systematic molecular analysis, e.g., the investigation of substitution peptide libraries for EPANPSEKNSPSTQY [45], which enable the replacement of each amino acid by alternative residues and identification of antibody binding mechanisms (Figure 1).

Peptide arrays have become powerful platforms for studying tumor signaling complexity, drug–target engagement, and adaptive resistance mechanisms in near-physiological environments. Recently, the quantitative and functional resolution of peptide microarrays in tumor systems biology has improved significantly [32]. Modern silicon-based high-density arrays now support >10^6^ peptides per assay, enabling simultaneous profiling of kinase activities, receptor–ligand interactions, and autoantibody repertoires across multiple cancer types with a statistical power sufficient for disease classification and presymptomatic immune monitoring [30]. High-throughput profiling of plasma from early-stage lung adenocarcinoma patients revealed autoantibody signatures with >85% sensitivity and 90% specificity, outperforming conventional imaging alone and providing prognostic insight post surgery [46]. Quantitative mapping of post-translationally modified and non-canonical peptides across tumor immunopeptidomes demonstrates that 25–40% of presented antigens are not predictable from transcriptomic data, highlighting the unique functional information captured by peptide arrays [47]. The integration of microfluidics has further advanced the biological relevance of these systems [48], enabling high-throughput kinetic measurements, low-volume control, and dynamic signal capture that static spotted slides cannot achieve [49]. Closed-system microfluidic peptide chips achieve picoliter-scale reactions with active sample recirculation, leading to enhanced binding kinetics, improved signal-to-noise ratios, and reduced assay variability, while requiring orders of magnitude less sample input than conventional formats, which is a critical advantage for precious tumor lysates and clinical specimens [50,51]. Controlled flow and dynamic assay formats permit real-time monitoring of kinase kinetics, ligand binding, and drug-induced signaling rewiring. Microfluidic peptide platforms have also been applied to physiologically relevant activity mapping, such as integrated detection of tyrosine kinase auto-phosphorylation in enzymatic networks [52], confirming that membrane context and microenvironmental factors quantitatively alter catalytic activity on-chip, bridging in vitro biochemical behavior with contextual mechanistic insight. In parallel, microfluidic peptide chips are capable of detecting cell invasion mechanisms of metastatic breast cancer cells and their interplay with endothelia cells [48]. The arrays present thousands of matrix-derived and modified peptide motifs [32], enabling mapping of cell–matrix interactions and adhesion phenotypes [48]. This can provide functional parameters relevant to invasion and metastasis that complement transcriptomic and proteomic profiles.

**Figure 2:**
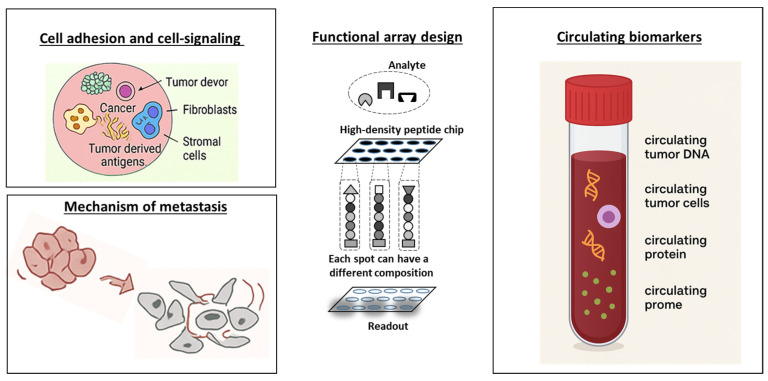
Overview of the setup of a peptide chip and its applications.

Meanwhile, high-density peptide arrays provide thousands of capture molecules, like extracellular matrix (ECM)-derived fragments, to screen for peptides that modulate cancer cell adhesion [32] or reveal novel regulators of cell–matrix interactions [14]. One important point is the design of the library: To build the peptide library for the chip, one can first derive candidate peptide sequences from resources such as PeptideAtlas [53] and then use solid-phase peptide synthesis, followed by purification and quality control. For pre-synthesized libraries, peptides are typically spotted onto functionalized supports (e.g., NHS-ester glass slides) using robotic microarray printers [54]. The printing buffer and linker or carrier (e.g., biotin–streptavidin, or PEG-linker) can influence the correct orientation and stable immobilization [54]. Coupling peptide arrays with information from complementary technologies like mass spectroscopy [55], CRISPR-based peptide libraries with AI analysis [56], or multi-omics datasets [57], we can investigate a large research field from discovery to precision medicine. Although CRISPR and AI approaches can enhance peptide array analyses for kinase–target identification, potential biases towards understudied kinases may be considered. AI-driven analysis [58,59] has enabled hierarchical data integration from molecular interaction landscapes to phenotypic outputs, facilitating tumor antigen landscape identification, dynamic signaling network rewiring, and drug resistance mechanism elucidation across scales [60,61]. Collectively, these advances position peptide arrays as quantitative oncological platforms that extend beyond static binding assays to interrogate in situ kinetics and regulatory networks under near-physiological conditions [62]. Here, we give an overview on peptide array applications for cell-based assays, tumor tissue analysis, blood-based assays, drug screening, and the discovery of mechanisms of drug resistance (Figure 2).

### 2.1. Cell-Based Assays: Mapping Adhesion, Migration, and Signaling

Tumor-associated changes in cellular communication can shape angiogenesis, metastasis, and tumor immune evasion. Among the different cellular components of the tumor microenvironment, endothelial cells and macrophages emerge as key regulators of tumor progression [63,64,65]. Tumor endothelia cells [66] influence immune cells and immune-checkpoint pathways due to immunomodulatory ligands [63]. In parallel, macrophages adapt to their local microenvironment [66] and promote immune suppression and liver metastases by various mechanisms [67]. Peptide chips further illuminated the endothelial–macrophage axis in tumor progression. Tie2-targeting peptides [68] enabled inhibition of Angiopoietin-mediated pro-angiogenic signaling in both endothelial cells and tumor-associated macrophages, and resulted in reduced vessel formation and limited tumor growth [69,70]. High-density peptide arrays facilitate the systematic identification of motifs controlling cell adhesion, migration, and signal transduction, which are critical determinants of metastasis and therapy resistance. Sonnentag et al. identified peptides regulating tumor cell clustering and motility [32], directly linking extracellular patterns to resistance mechanisms. A particularly impactful example is the work by Sonnentag et al. [18], which moved beyond descriptive screening by linking specific extracellular peptide motifs to functional phenotypes such as tumor cell clustering, migration, and repulsion. By systematically correlating peptide–cell interactions with downstream cytoskeletal reorganization and migratory behavior, this study demonstrated how short extracellular motifs can actively shape resistance-associated phenotypes. Importantly, the work illustrates a key strength of peptide arrays: the ability to connect molecular binding events with emergent cellular behavior under controlled conditions [32]. This level of mechanistic insight is difficult to achieve with other approaches, e.g., flow cytometry would require the analysis of single cells in suspension and microscope assays would still require the functionalized surface and cannot be used as a standalone technique. The authors identified endothelia cell motifs that enhance adhesion and angiogenic spreading by vascular remodeling supporting tumor survival [71]. Kinome-focused arrays extend this analysis to cell lysates, capturing intracellular adaptations [72]. Cellular interactions are very complex [73,74], especially within the tumor microenvironment, which can lead to immune cell exhaustion and altered cellular composition by metabolic reprogramming or mitochondrial dysfunction [75]. Furthermore, tumor-associated ECM remodeling and cellular interaction with ECM molecules can drive tumor progression by influencing pathway crosstalk [73,74,76]. Peptide arrays allow the systematic screening of extracellular motifs that regulate cell–matrix interactions, as shown by Kanie et al. [77]. The authors used a SPOT peptide microarray to identify tripeptides selectively binding collagen IV. Building on this platform, automated microtiter-plate synthesis of (phospho)peptide arrays enables efficient probing of commercially available kinase substrates which play an important role in intracellular signaling and could be further developed to investigate cellular responses [78].

However, surface-immobilized short motifs can lead to misleading cell interactions that depend normally on the full-length protein binding [15]. Furthermore, surface chemistry, ligand density, and molecular orientation can change affinity and cell adhesion strength. Another limitation is the nonspecific adsorption of cells, which can result in strong signal background or incorrect data especially for complex samples composed of multiple cell types [79]. These limitations need to be taken into account for data interpretation.

### 2.2. Blood-Based Assays: Detecting Circulating Biomarkers

Peptide arrays also support high-throughput profiling of circulating molecules directly from blood (Figure 2), including tumor-associated antigens [49], autoantibodies [80,81,82,83], neoantigens with more than 400,000 peptides [49,84], and low-abundance proteins, offering minimally invasive windows into systemic tumor–host interactions [85]. Electrochemical peptide arrays can detect femtomolar concentrations of tumor peptides from microliter-scale serum samples, achieving sensitivity comparable to ELISA [85]. Iyer et al. [86] and Cao & Chen [87] demonstrated that miniaturized lab-on-chip systems integrate sample routing, multiplexed detection, and automated analysis, enabling point-of-care testing. Peptide arrays also provide functional insights into signaling dynamics. Studies showed that arrays map SH2 and SH3 interactions and phosphorylation events, revealing ligand accessibility and conformational changes induced by growth factors or oncogenic signaling [88,89,90]. However, reproducing the full complexity of tumor microenvironments, cellular heterogeneity, and extracellular matrix dynamics remains a limitation. Furthermore, early detection of cancer remains a critical challenge in oncology, as tumors are often asymptomatic in initial stages, limiting the effectiveness of current imaging-based screening approaches. Tumor-associated autoantibodies (TAAbs) arise in the patient’s blood years before clinical manifestation, serving as sensitive biosensors of early tumorigenesis [90,91,92,93,94,95,96,97,98,99,100,101,102,103]. These autoantibodies can provide non-invasive, blood-based biomarkers that complement existing imaging modalities, improving early diagnosis and patient outcomes (Table 1).

High-density peptide arrays have been developed for profiling autoantibody repertoires. In landmark studies, arrays containing over 130,000 synthetically synthesized peptides, representing mimotopes of tumor-associated antigens, were used to capture autoantibodies from plasma of early-stage lung adenocarcinoma patients [91]. The cohorts included 377 samples, enabling systematic identification of disease-specific immune signatures. Notably, when combined with low-dose computed tomography, autoantibody profiling improved the positive predictive value from 50% to 78.3%, demonstrating how immunoprofiling can complement imaging [91]. Beyond predictive accuracy, peptide arrays showed higher sensitivity than conventional tumor markers, detecting 72–81% of cases versus 22% for markers such as CYFRA21.1, NSE, SCC, and ProGRP. This sensitivity highlights their potential to identify tumors at an earlier, clinically actionable stage [91]. The study by Luo et al. [109] represents a conceptual advance in blood-based cancer diagnostics by demonstrating that autoantibody signatures derived from large-scale peptide libraries can outperform conventional tumor markers in sensitivity and temporal resolution. Notably, the technique significantly improved the predictive value, which can be clinically relevant. This work highlights how peptide arrays can capture early immune surveillance events that precede radiologically detectable disease, positioning them as promising tools for population-level screening strategies when combined with established imaging modalities [109]. High-density peptide arrays now provide a scalable platform for mapping disease-specific autoantibody repertoires, supporting early diagnosis, patient stratification, and integration into precision screening workflows. However, careful validation and replication are essential to minimize false-positive signals, particularly in low-prevalence cohorts [110].

### 2.3. Tumor-Sample-Based Methods

Tumor progression and therapeutic resistance are shaped by complex interactions among cancer cells, stromal cells, and immune components, as well as by extracellular matrix remodeling and nutrient gradients [111,112,113,114]. Peptide arrays are a powerful tool to identify tumor binding peptides, screen tumor cells, and profile immune reactions [46,88]. Key molecular and cellular determinants include cell adhesion motifs, migration regulators, angiogenic signals, cytokine secretion, and kinase–substrate networks [2,115]. Accurate detection and functional mapping of these factors are essential to understand how tumors adapt, evade therapy, and metastasize. High-throughput peptide screens have revealed motifs regulating tumor cell adhesion, migration, and clustering, directly linked to metastatic potential and therapy resistance [32,116]. To systematically interrogate these determinants, researchers have employed high-density peptide arrays [38] and tumor-on-a-chip systems. Peptide arrays provide direct readouts of extracellular ligand–receptor interactions and motif-specific signaling [32]. These arrays capture dynamic cell–cell and cell–matrix interactions under controlled microenvironmental conditions, offering mechanistic insights that are difficult to obtain with traditional techniques. The method can be combined with CODEX [117,118], spatial transcriptomics [119], or single-cell sequencing [120], which often provide static snapshots rather than functional activity. Integration of spatial transcriptomics and single-cell profiling complements peptide array analysis, illuminating the spatial–functional organization of immune subsets such as macrophages and NK cells within tumors [120].

#### 2.3.1. Multi-Omics and Computational Integration: From Motifs to Mechanistic Models

Integration with multi-omics data and computational modeling is expected to enhance predictive power and accelerate adoption in early cancer detection. Zhang et al. [121] demonstrated that integrating array-based readouts with proteomic datasets identifies cancer-type-specific signaling signatures, highlighting tumor heterogeneity. Integration of peptide array data with transcriptomics, proteomics, and AI-driven modeling [122] enables prediction of kinase–substrate interactions [115], tumor-specific epitopes, and signaling network rewiring [123]. Integration with multi-omics datasets [124,125] further enhances mechanistic insight. Coupling arrays with mass spectrometry [55], microchip electrophoresis, and deep learning models predicts kinase–substrate relationships and pathway rewiring under perturbations such as drug treatment, immune activation, or metabolic stress [126,127]. Computational modeling has enabled patient-specific signaling reconstruction [121]. Coupling peptide-binding landscapes with gene expression and post-translational modifications provides a multimodal view of tumor biology, accelerating biomarker discovery, immune epitope mapping, and characterization of tumor heterogeneity. Novel studies [123,128] showed that network-based models identify unique protein subnetworks in individual tumors, guiding tailored therapeutic strategies. Peptide arrays are designed to probe functionally relevant regions of the proteome. The PeptideAtlas resource [53] plays a central role in this process, as it compiles high-confidence, experimentally observed peptides across multiple proteomes, including post-translational modifications. By leveraging this repository, researchers can select biologically validated peptide sequences for array synthesis, ensuring coverage of disease-relevant proteins and motifs. This strategy is particularly valuable for neoantigen discovery [11] and personalized cancer immunotherapy, where arrays must capture patient-specific antigenic determinants to evaluate immune recognition efficiently. A key challenge remains the interoperability of peptide array data with other omics layers, such as transcriptomics and proteomics [129,130]. Another limitation is the 3D structure and multicellular composition [131].

#### 2.3.2. Drug Screening

Peptide arrays provide a functional platform for targeted drug discovery and resistance mechanism mapping. Screening peptide–drug interactions identifies binding epitopes and pathways contributing to therapy failure. Microfluidic metastasis-on-chip studies [132] were performed. Those experiments can show that fibroblast-driven inflammation and ECM remodeling enhances invasion. Differences in the ECM molecule composition, such as collagen I, can drive angiogenesis and cancer progression [133]. These examples illustrate the functional detection of cellular behavior and therapeutic vulnerability. Compared to conventional spatial or single-cell approaches, peptide arrays provide high-resolution, functional interrogation of extracellular motifs, allow direct testing of drug response and signaling adaptation, and can quantitatively link molecular signatures to multicellular behavior. However, peptide arrays cannot fully capture the three-dimensional organization, mechanical properties, or dynamic gradients of the tumor microenvironment and soluble biomarkers, which are critical for drug response [134]. Another issue might be low binding events, which could be washed away during sample processing.

## 3. Discussion

**Figure 3:**
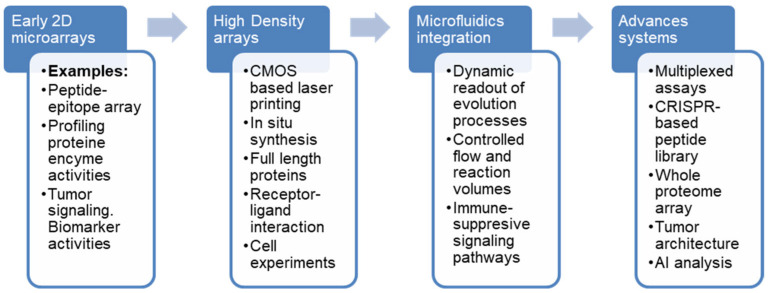
Technological advances of peptide arrays [40,43,44,49,56,57].

Collectively, the studies discussed in this review indicate that peptide microarrays have evolved from exploratory screening tools into hypothesis-generating and mechanism-resolving platforms [39,50,109,122]. Their main conceptual strength lies in the direct interrogation of functional molecular interactions, enabling insights into signaling plasticity, microenvironmental adaptation, and therapy resistance. However, the interpretation of peptide array data requires careful consideration of biological context [39], as linear peptides may not fully recapitulate conformational epitopes or higher-order protein structures [30]. Thus, peptide arrays are most powerful when embedded within integrative experimental workflows rather than used as standalone discovery tools. Peptide microarrays have emerged as versatile analytical platforms that enable high-resolution insights into tumor biology, offering unique opportunities to dissect molecular mechanisms underlying metastasis and cell migration. The technology has made significant progress during the last decade (Figure 3), enabling a broad biological application to answer tumor-related questions.

Firstly, peptide chips enable the subsequent analysis of parts of a protein and the effect on drug or antibody binding affinities, which are difficult to study with other techniques. The native biological function and effect of molecular architecture can be investigated for short motifs as well as whole proteins [45,82]. Those proteome arrays serve as high-throughput tools for analyzing the target architecture of antibodies and cellular receptors. Through the targeted isolation of low-molecular-weight proteins, authors such as Brouwers et al. [135] identified distinct protein signatures between metastatic and benign tumors, supporting early detection and improved prognosis [136,137]. However the experimental approaches are limited by linear peptides, nonspecific binding, and underrepresentation of understudied targets, necessitating orthogonal validation [138].

Secondly, the combination with complementary techniques broadens the scope of applications. Integrating peptide chips with high-resolution mass spectrometry allows spatial mapping of biomarkers and extracellular interactions within tumor lesions [139,140]. However, for investigation of gene expression patterns at cellular resolution, spatial transcriptomics is commonly used [119,120].

Thirdly, peptide chip-based dynamic studies are performed, e.g., for investigation of cell migration, which can reveal key signaling pathways, such as integrin, VEGFR, or CXCL12/CXCR4, that promote cell migration and the epithelial–mesenchymal transition [141]. Further studies demonstrated that functional on-chip assays yield quantitative information about cell motility, enabling the identification of potential therapeutic targets [32]. Combinations with microfluidic and metastasis-on-chip models further revealed that the interaction between tumor and stromal cells actively governs invasion and extracellular matrix remodeling, while MEK inhibitors such as imatinib stabilize the endothelial barrier and inhibit intravascular migration [142,143]. Overall, the findings confirm that peptide chips provide mechanistic insights into metastasis and tumor progression and peptide-based cell selection to functional migration assays. Beyond descriptive profiling, the integration of peptide arrays with microfluidic control and computational modeling enables interrogation of binding kinetics, force-dependent interactions, and context-specific signaling responses under near-physiological conditions [49,50,86]. In combination with machine learning-based analysis [60,61,144], these high-dimensional datasets allow hierarchical integration of molecular interaction patterns with functional phenotypes, supporting the identification of metastasis-associated antigens, adaptive signaling rewiring, and therapy-induced resistance mechanisms. Consequently, peptide chip technologies are evolving to quantitative systems-biology platforms [49,132]. They complement classical omics approaches and open translational potential, for example, for personalized therapies or CAR-T strategies targeting metastasis-associated antigens [145]. By translating subtle immune recognition patterns into measurable diagnostic signatures, these platforms bridge molecular profiling and clinical oncology. To realize clinical translation, challenges such as standardization of peptide selection, array fabrication, and data interpretation, as well as validation in larger, diverse patient cohorts, need to be addressed. To ensure robust conclusions bias, study quality should be assessed, for example, using tools such as ROBIS, which evaluates the risk of bias across multiple methodological domains [146]. For investigation of cross-reactivity, key variables governing peptide array performance and reproducibility include peptide length and density [147], orientation/linkers, surface chemistries (e.g., NHS-ester, epoxide, aldehyde), blocking buffers, sample type and handling, dynamic range, replicate design, lot-to-lot variability, normalization, and FDR control [148,149]. For all experiments, comparative data, quantitative evidence for hit quality or in vivo predictivity, and detailed consideration of clinical translation should be validated carefully with orthogonal techniques. We suggest combining peptide-array-based profiling with complementary techniques [150,151,152,153] to validate tumor–stroma and tumor–immune interactions, thereby enhancing our understanding of tumor heterogeneity and therapeutic response [151]. Recently, Qian et al. [154] demonstrated mitochondria-targeting theranostic platforms as a promising complementary approach to peptide-array-based profiling, enabling targeted delivery and real-time monitoring of therapeutic efficacy at the subcellular level. This will help in designing personalized optimal treatments [155]. However, peptide microarrays are often limited by linear peptides, nonspecific binding, target underrepresentation, and design variability, and require validation and standardization for robust biological and clinical use.

## 4. Conclusions and Future Perspectives

Future developments in peptide array technology are expected to converge on several interrelated directions that collectively enhance biological relevance and translational impact. Integration of peptide arrays with three-dimensional culture systems and organ-on-chip models will enable functional interrogation of multicellular tumor niches under physiologically meaningful conditions, as microfluidic tumor-on-chip platforms have emerged as advanced systems for modeling tumor–ECM interactions, drug response, and resistance mechanisms beyond conventional two-dimensional cultures [26,132,151]. Overall, peptide chips provide a high-resolution platform to dissect tumor-stroma cell crosstalk, the epithelial–mesenchymal transition, and metastatic progression. They bridge molecular and functional phenotypes, offering actionable insights for therapy. While challenges such as standardization, reproducibility, and clinical validation remain, the technology complements existing omics approaches and advanced models. Integration of peptide chips with microfluidics, spatial proteomics, CRISPR screens, and AI-driven peptide design accelerates personalized anti-metastatic strategies. AI-driven peptide library design and advanced data analysis approaches are poised to accelerate the identification of context-specific binding motifs [156], resistance-associated interaction signatures [157], and patient-tailored therapeutic targets [144]. Recent advances in machine learning-based peptide design demonstrate that computational frameworks can efficiently explore large peptide sequence spaces [158,159]. These screens hold promise for integration into precision oncology workflows. Future studies should address ethical and translational hurdles, including AI-driven data analysis of peptide array technologies, 3D models, and steps toward clinical translation, such as patient validation and therapeutic implementation.

## Figures and Tables

**Table 1:** Overview of autoantibody detection techniques.

Cancer Type	Autoantibody Detection Technique
Lung adenocarcinoma	High-density peptide microarray profiled plasma to identify peptide autoantibody signatures [91]
Lung cancer (general)	Protein array and ELISA validation screening of cancer-driver proteins, developed 7-TAAb decision-tree panel [92]
Pancreatic cancer	Engineered glycopeptide probes (not array) peptide–antibody confirmed by SPR [93]
Pancreatic ductal adenocarcinoma	High-throughput protein microarrays screened sera to identify an 11-autoantibody panel [94]
Colorectal cancer	Protein-array workflow for serum screen of autoantibody signatures [95]
Melanoma (mouse model)	Whole-proteome high-density peptide array epitope mapping in mice [96]
Glioblastoma	Peptide microarray assessing IgG/IgM autoantibodies [97]
Renal cancer	Human proteome microarray to examine the differences in IgG and IgM autoantibodies in sera [98]
Prostate cancer	High-throughput protein arrays [99]
Colon cancer	High-density peptide microarrays for detection of autoantibody biomarkers of colon cancer [100]
Ovarian Cancer	Protein microarray to evaluate autoantibodies and tumor-associated antigens (GNAS, NPM1, p53) [101]
Breast cancer	High-density protein microarrays were functionalized with 4988 candidate tumor antigens of patients with early-stage breast cancer and IgG [102]
Hepatocellular carcinoma	Human Proteome Microarray was used to detect autoantibodies to a panel of six tumor-associated antigens (RAD23A, CAST, RUNX1T1, PAIP1, SARS, PRKCZ) [103,104]
Hepatitis B-related hepatocellular carcinoma	Proteome microarrays enabled the detection of autoantibodies against tumor-associated antigens (TAAbs) and a candidate biomarker panel (APEX2, RCSD1, and TP53). [105,106]
Alveolar rhabdomyosarcoma	Protein microarray screens found PCDHGC5 autoantibodies as an independent negative prognostic factor and ARMS as a marker for immune response [107]
Soft tissue sarcoma	Serological analysis of recombinant cDNA expression libraries was used to generate a list of tumor-associated antigens as potential biomarkers and therapy targets (DLG7, JUN) [108]

## Data Availability

Data are available upon request from anna.grab@umm.de.
